# Supplementary material for: Histone H3.3 deposition in seed is essential for the post-embryonic developmental competence in Arabidopsis
Source: Nat Commun. 2022 Dec 13;13:7728. doi: 10.1038/s41467-022-35509-6 (PMC9747979; doi:10.1038/s41467-022-35509-6)
Supplement: Supplementary file 1 — Supplementary Information [file 41467_2022_35509_MOESM1_ESM.pdf]

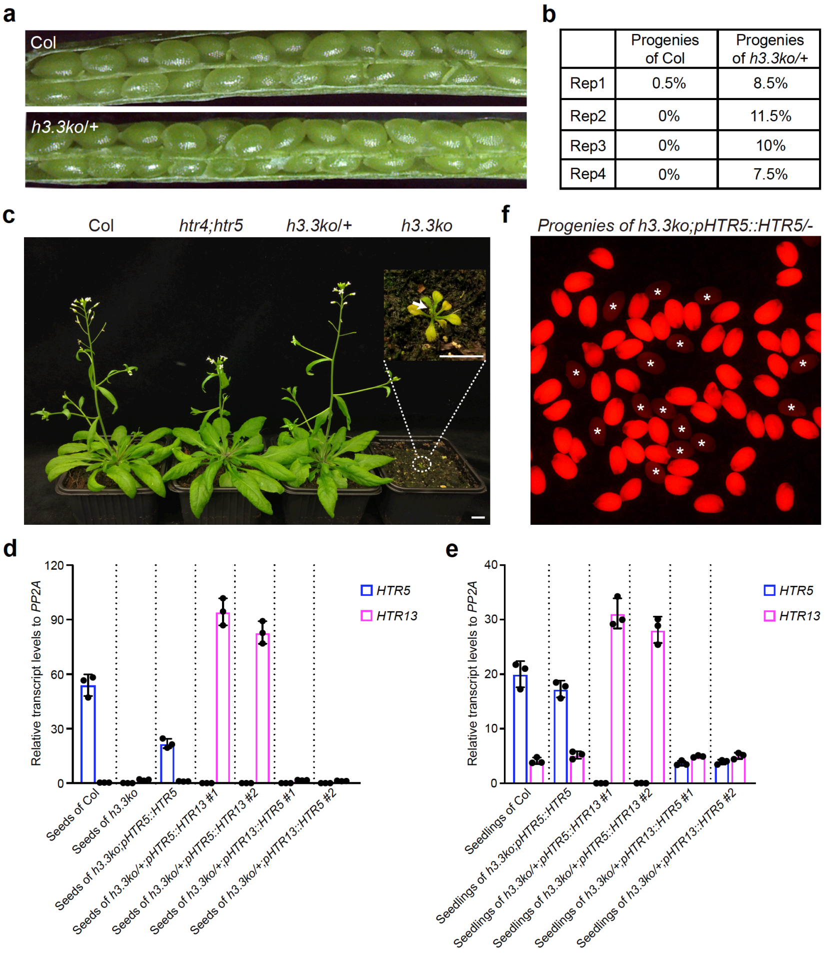

Supplementary Figure 1. *h3.3ko* phenotypes and expression analysis of HTR5 and HTR13 in transgenic lines.

a. Seed development of Col and *h3.3ko/+* (*htr4/htr4;htr5/htr5;htr8/+*).

b. Percentages of ungerminated seeds after 7 days imbibition, 200 seeds were scored in each replicate.

c. The developmental phenotype of Col, *htr4/htr4;htr5/htr5*, *h3.3ko/+* (*htr4/htr4;htr5/htr5;htr8/+*) and *h3.3ko* (*htr4/htr4;htr5/htr5;htr8/htr8*) at bolting stage. An inflorescence-like structure on *h3.3ko* is indicated with an arrow. Scale bars=1cm.

d and e. Relative transcript levels of *HTR5* and *HTR13* in the mature seeds (d) or 7-day-old seedlings (e) of indicated lines. The *HTR5* amplification primers were designed to only amplify the WT but not Crispr-mutated *HTR5.* Primers amplifying *HTR5* and *HTR13* were tested to have similar amplification efficiency. Values are means ± SD of three biological replicates. *PP2A* was used as an endogenous control for normalization.

f. Selection of *h3.3ko* seeds from *h3.3ko;pHTR5::HTR5/-* progenies. *h3.3ko* seeds without the complementation construct (mCherry signals) are indicated with asterisks.

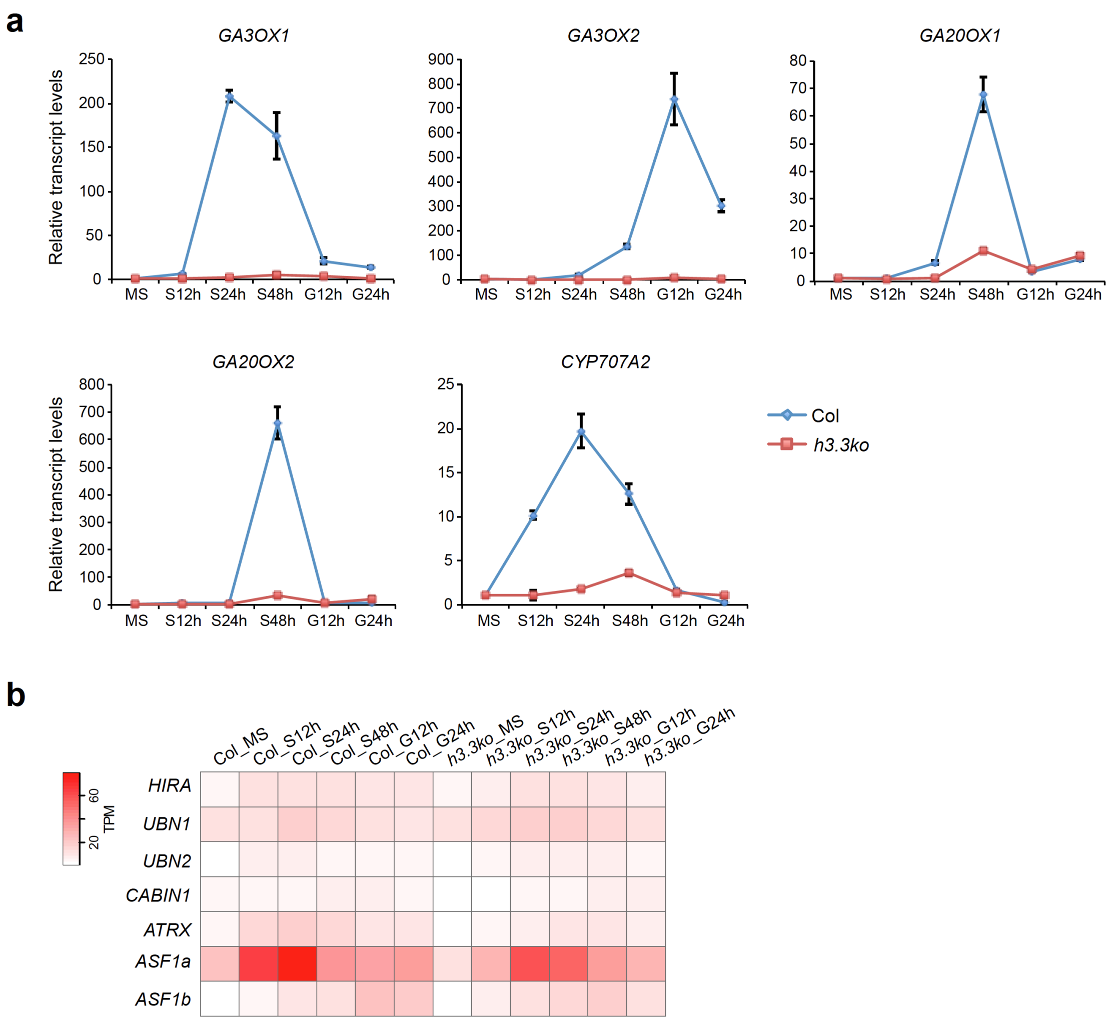

Supplementary Figure 2. Gene expression analysis during imbibition in Col and *h3.3ko*.

a. Transcript levels of *GA3OX1*, *GA3OX2*, *GA20OX1*, *GA20OX2* and *CYP707A2* during imbibition determined by RT-qPCR in Col and *h3.3ko*. Values are means ± SD of three biological replicates. *PP2A* was used as an endogenous control for normalization.

b. Heatmap showing the expression of H3.3 deposition chaperones during imbibition determined by RNA-seq in Col and *h3.3ko*. Values are means of three biological replicates.

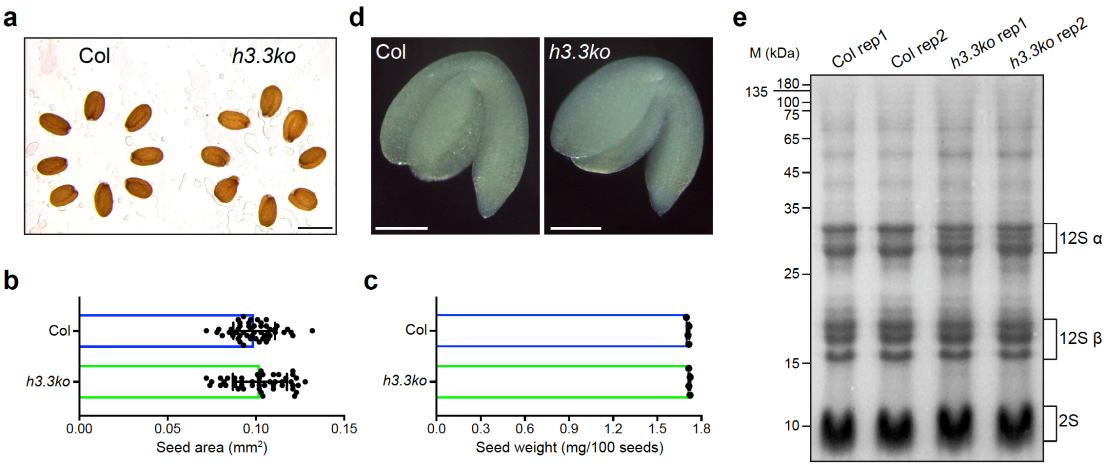

Supplementary Figure 3. *h3.3ko* embryo is morphologically intact.

a. Representative mature seeds of Col and *h3.3ko*. Scale bar=0.5mm. Three independent experiments were performed with similar results.

b and c. Seed size (b) and 100-seed weight (c) of Col and *h3.3ko*. 50 and 47 mature seeds were measured for the size of Col and *h3.3ko* respectively. Four replicates were measured for seed weight. Values are means ± SD.

d. Representative mature embryo of Col and *h3.3ko*. Scale bars=0.1mm. Twenty mature embryos of Col or *h3.3ko* were dissected with similar results.

e. Total proteins extracted from the same number of Col and *h3.3ko* mature seeds. 12S α and 12S β globulins and 2S albumins were indicated. Three independent experiments were performed with similar results.

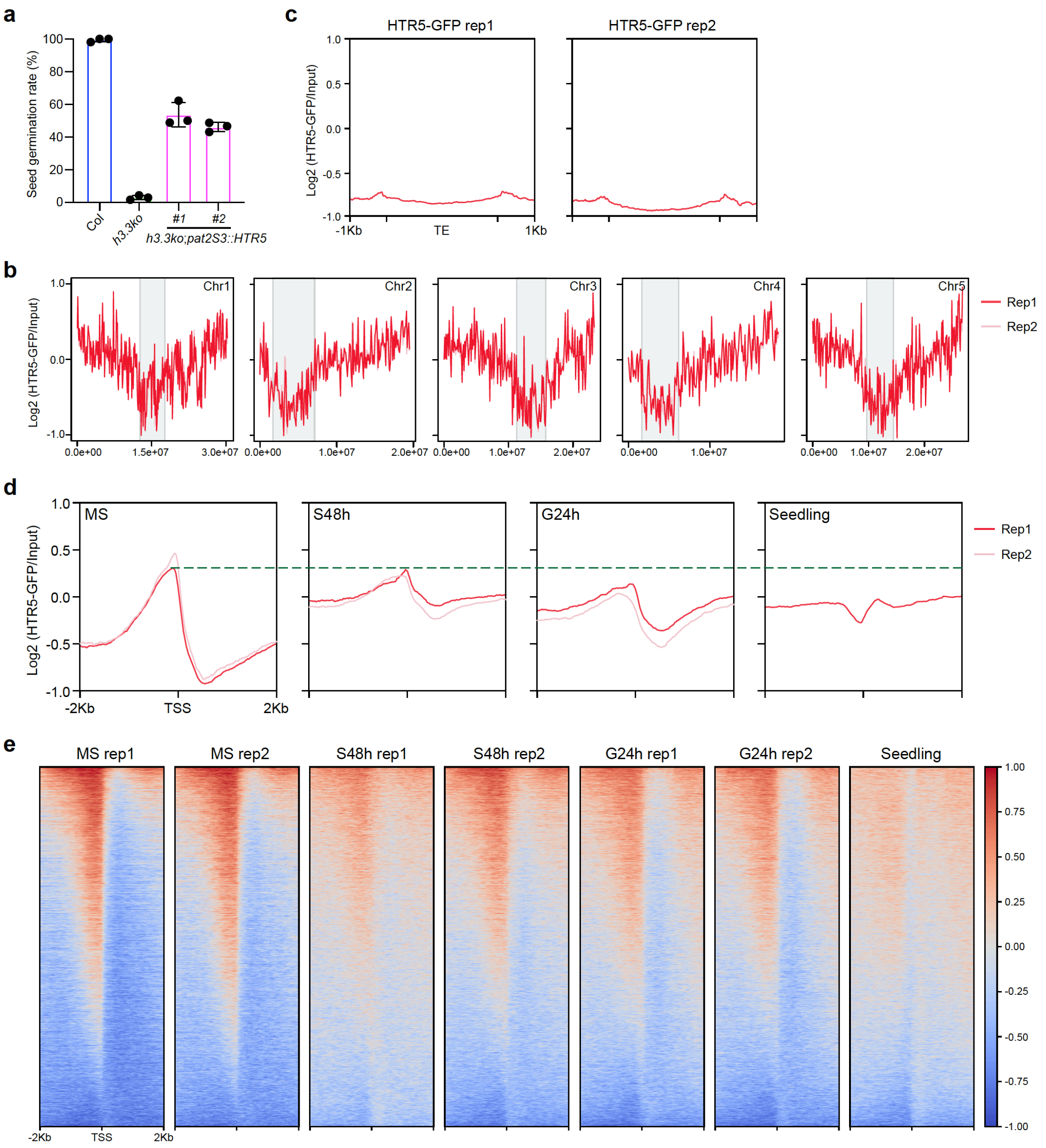

Supplementary Figure 4. HTR5-GFP distribution in mature seeds.

a. Percentages of *h3.3ko;pat2S3::HTR5* seeds that germinated within 1 month of imbibition. Values are means ± SD of three biological replicates. At least 40 seeds were assessed for each replicate.

b. HTR5-GFP ChIP-seq signals in mature seeds over *Arabidopsis* chromosomes. Signals were calculated in 100kb bins. Pericentromeric heterochromatin regions are indicated with grey shading.

c. Metaplot of HTR5-GFP ChIP-seq signals in mature seeds over TEs.

d. Metaplot of HTR5-GFP ChIP-seq signals over all TSS regions in mature seeds, during imbibition, and in the 10-day-old seedling.

e. Heatmap of HTR5-GFP ChIP-seq signals over all TSS regions in mature seeds, during imbibition, and in the 10-day-old seedling.

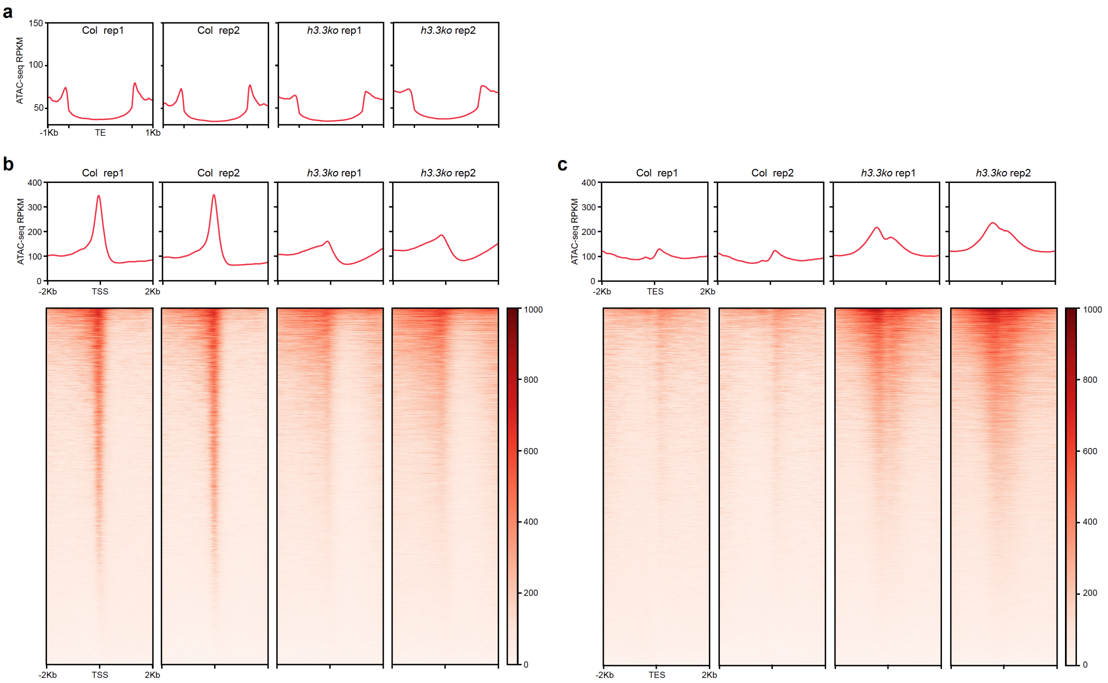

Supplementary Figure 5. Chromatin accessibility over TEs and genes in mature Col and *h3.3ko* seeds.

a. Metaplot of ATAC-seq signals in Col and *h3.3ko* mature seeds over TEs.

b. Metaplot and heatmap of ATAC-seq signals in Col and *h3.3ko* mature seeds over all TSS regions.

c. Metaplot and heatmap of ATAC-seq signals in Col and *h3.3ko* mature seeds over all TES regions.

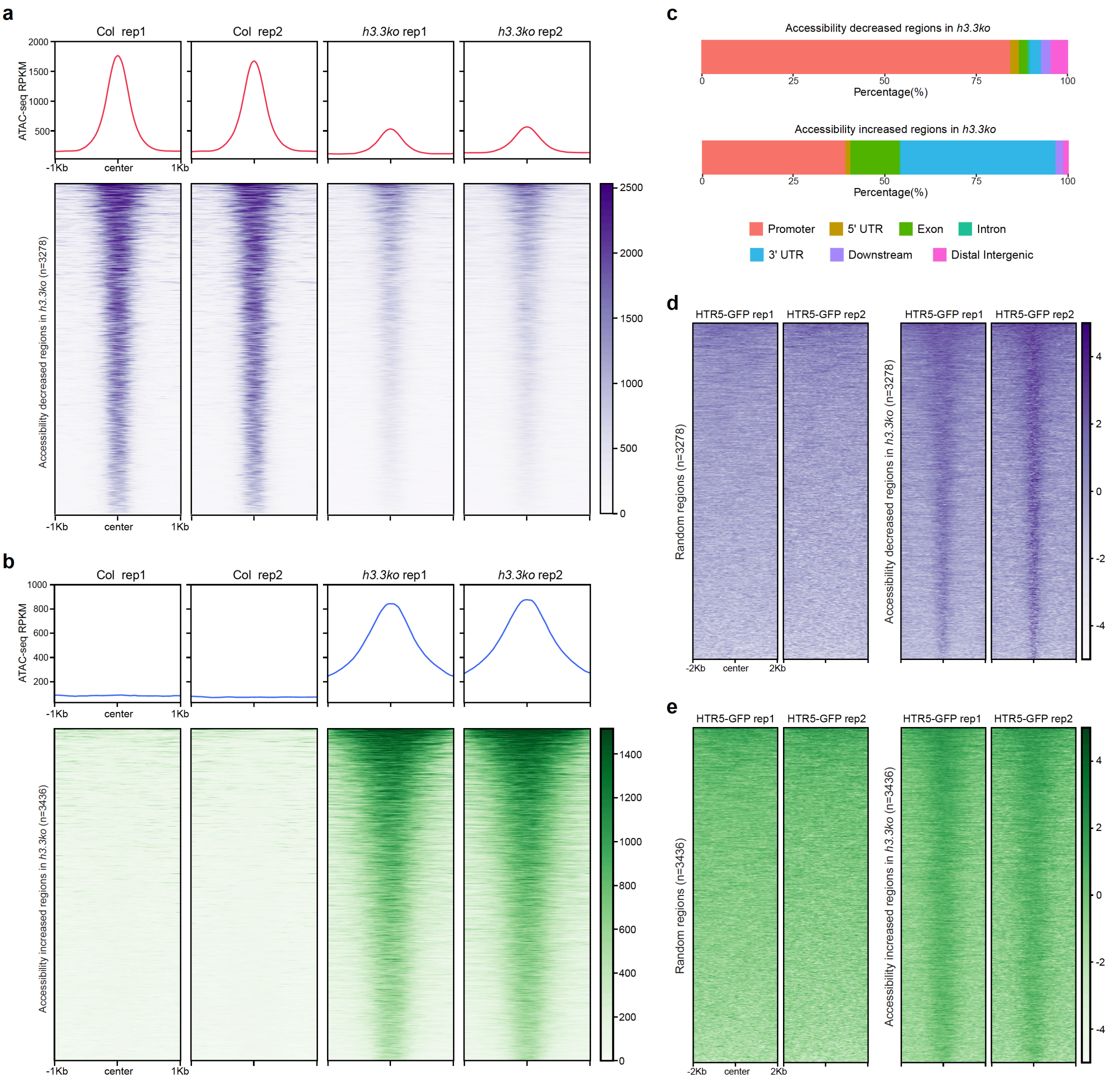

Supplementary Figure 6. Chromatin accessibility and HTR5-GFP enrichment over accessibility decreased and increased regions in mature *h3.3ko* seeds compared with Col.

a and b. Metaplot and heatmap of ATAC-seq signals in Col and *h3.3ko* mature seeds over accessibility significantly decreased (a) and increased (b) regions in mature *h3.3ko* seeds compared with Col.

c. Genomic distribution of accessibility significantly decreased and increased regions in mature *h3.3ko* seeds compared with Col.

d and e. Heatmap of HTR5-GFP signals in mature seeds over accessibility significantly decreased (d) and increased (e) regions in mature *h3.3ko* seeds compared with Col. HTR5-GFP signals over randomly selected regions are served as control.

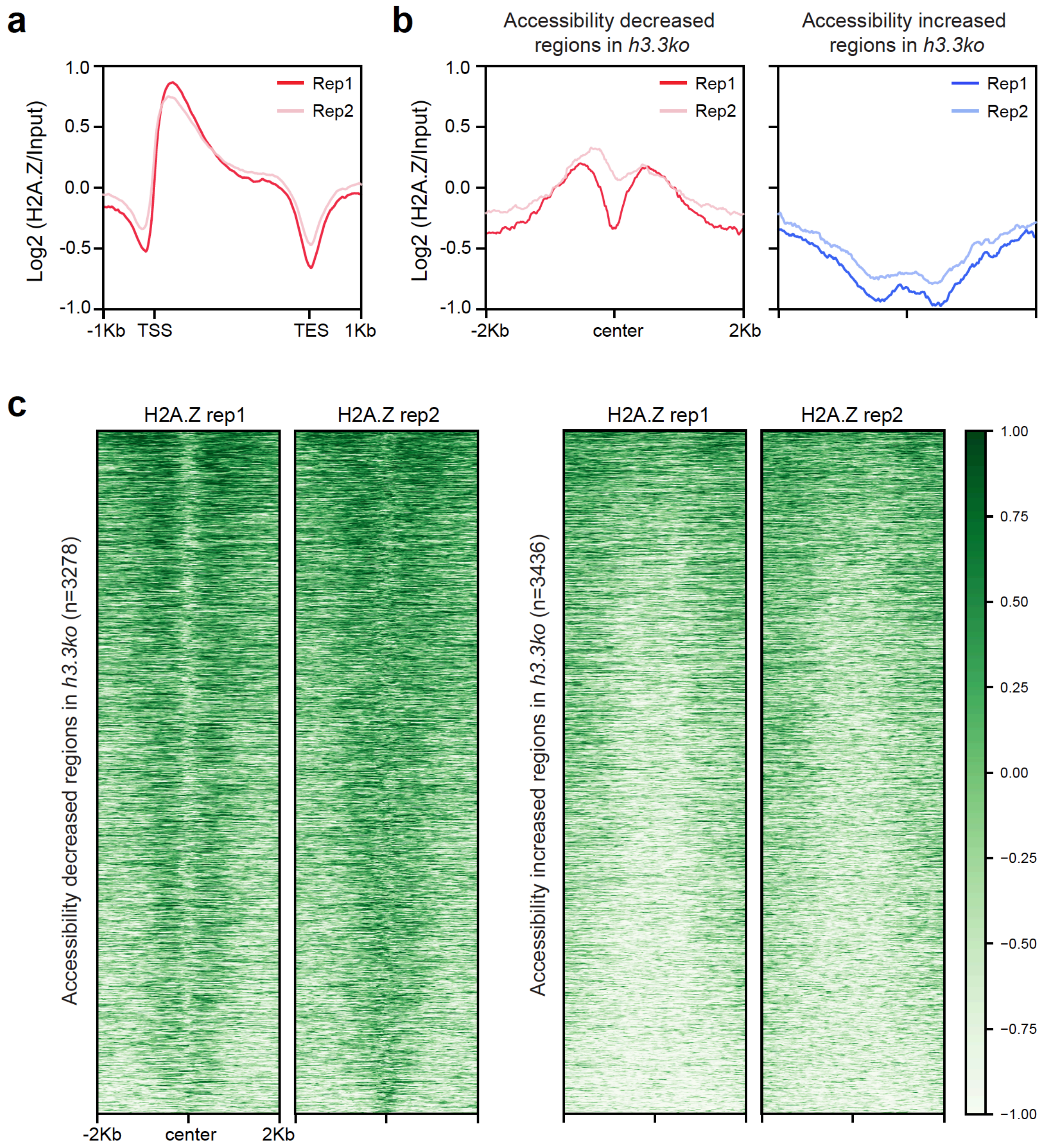

Supplementary Figure 7. H2A.Z distribution in mature seeds.

a. Metaplot of H2A.Z ChIP-seq signals in mature seeds over all genes.

b. Metaplot of H2A.Z ChIP-seq signals in mature seeds over accessibility significantly decreased and increased regions in mature *h3.3ko* seeds compared with Col.

c. Heatmap of H2A.Z ChIP-seq signals in mature seeds over accessibility significantly decreased and increased regions in mature *h3.3ko* seeds compared with Col.

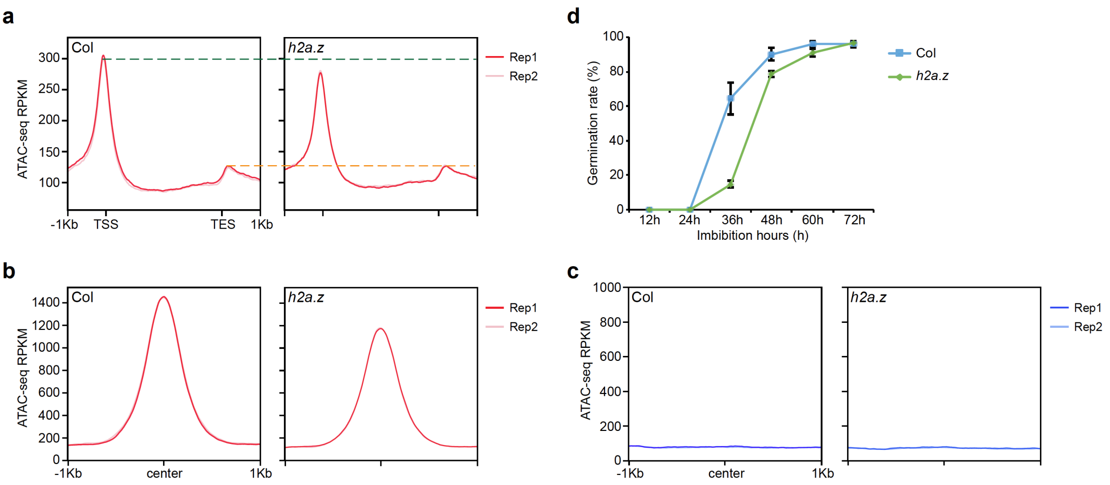

Supplementary Figure 8. Chromatin accessibility analysis in *h2a.z* mutant.

a. Metaplot of ATAC-seq signals in Col and *h2a.z* mature seeds over all genes.

b and c. Metaplot of ATAC-seq signals in Col and *h2a.z* mature seeds over accessibility significantly decreased (b) and increased (c) regions in mature *h3.3ko* seeds compared with Col.

d. Germination rates of Col and *h2a.z*. Values are means ± SD of three biological replicates. 196 seeds were assessed in each replicate.

a

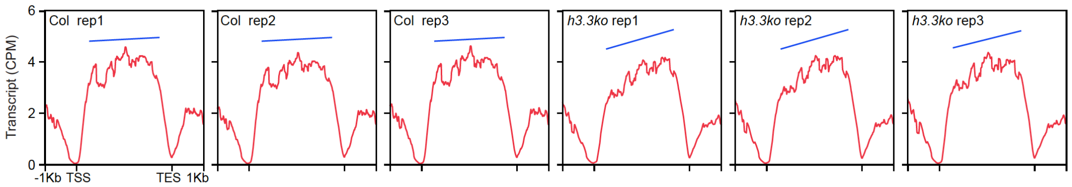

b

Bin position	*P*-value
1/50	0
2/50	4.37E-37
3/50	0.157108622
48/50	0.000497745
49/50	8.35E-32
50/50	5.95E-160

c

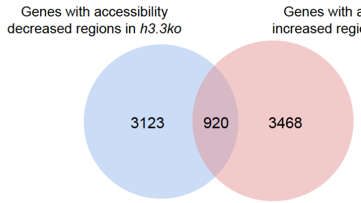

d

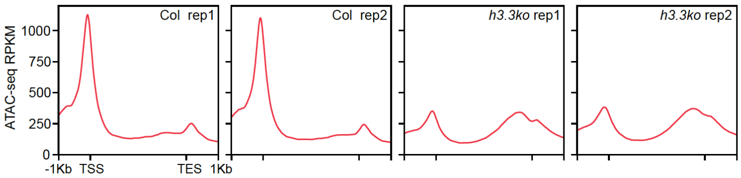

e

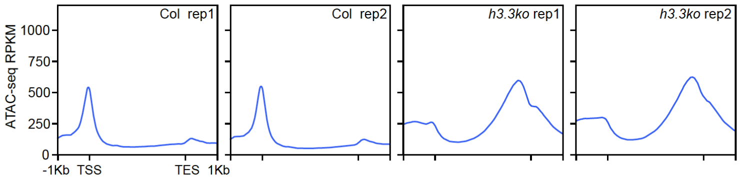

Supplementary Figure 9. Altered chromatin accessibility in *h3.3ko* affects transcription.

a. Metaplot of transcripts in Col and *h3.3ko* mature seeds over all genes in three biological replicates.

b. Significance analysis of difference between transcript levels in Col and *h3.3ko* mature seeds around the 5' and 3' gene ends. Transcripts on genes were divided into 50 bins from the 5' to 3'. *P*-values of the first (5') and last (3') three bins were shown, *P*-values were calculated with the two-sided Wilcoxon test.

c. Venn diagrams of accessibility decreased and increased region associated genes in mature *h3.3ko* seeds.

d and e. Metaplot of ATAC-seq signals in Col and *h3.3ko* mature seeds over genes with accessibility significantly decreased (d) and increased (e) regions in mature *h3.3ko* seeds.

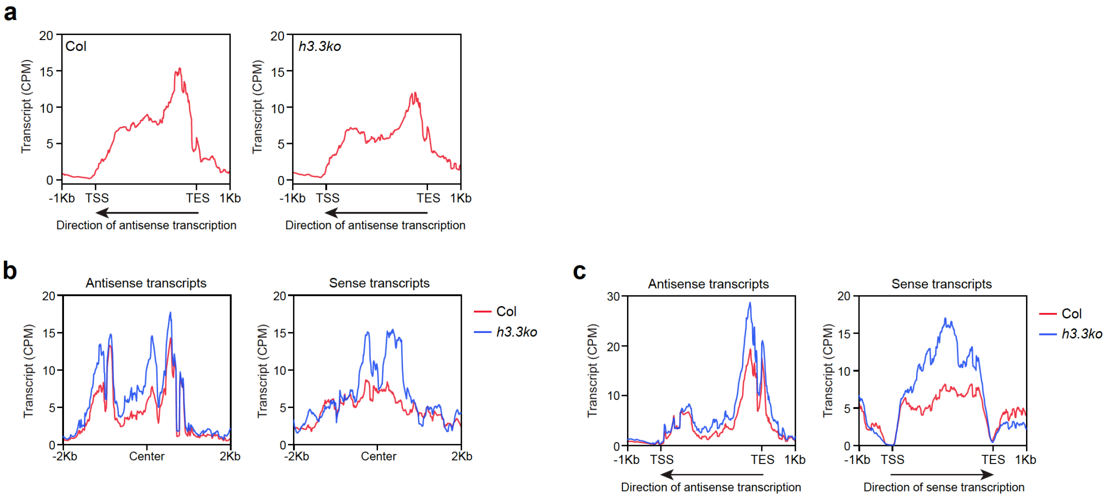

Supplementary Figure 10. Antisense transcripts analysis in mature *h3.3ko* seeds.

a. Metaplot of antisense transcripts in Col and *h3.3ko* mature seeds over all genes. The profiles were generated after merging three biological replicates.

b. Metaplot of sense and antisense transcripts in Col and *h3.3ko* mature seeds over accessibility significantly increased regions in mature *h3.3ko* seeds compared with Col. The profiles were generated after merging three biological replicates.

c. Metaplot of sense and antisense transcripts in Col and *h3.3ko* mature seeds over genes with accessibility significantly increased regions in mature *h3.3ko* seeds. The profiles were generated after merging three biological replicates.

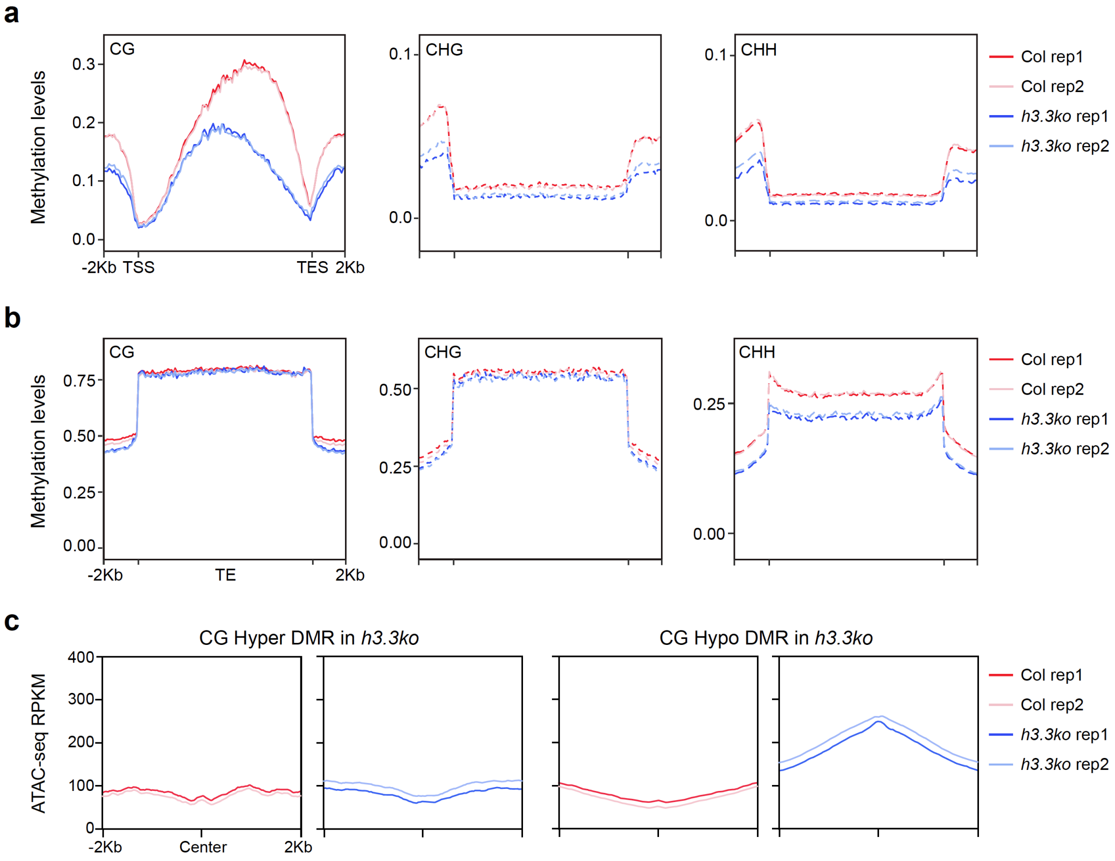

Supplementary Figure 11. DNA methylation changes in mature *h3.3ko* seeds.

a and b. Metaplot of DNA methylation levels in Col and *h3.3ko* mature seeds over all genes (a) or TEs (b).

c. Metaplot of ATAC-seq signals in Col and *h3.3ko* mature seeds over hyper and hypo CG methylation regions in mature *h3.3ko* seeds compared with Col.

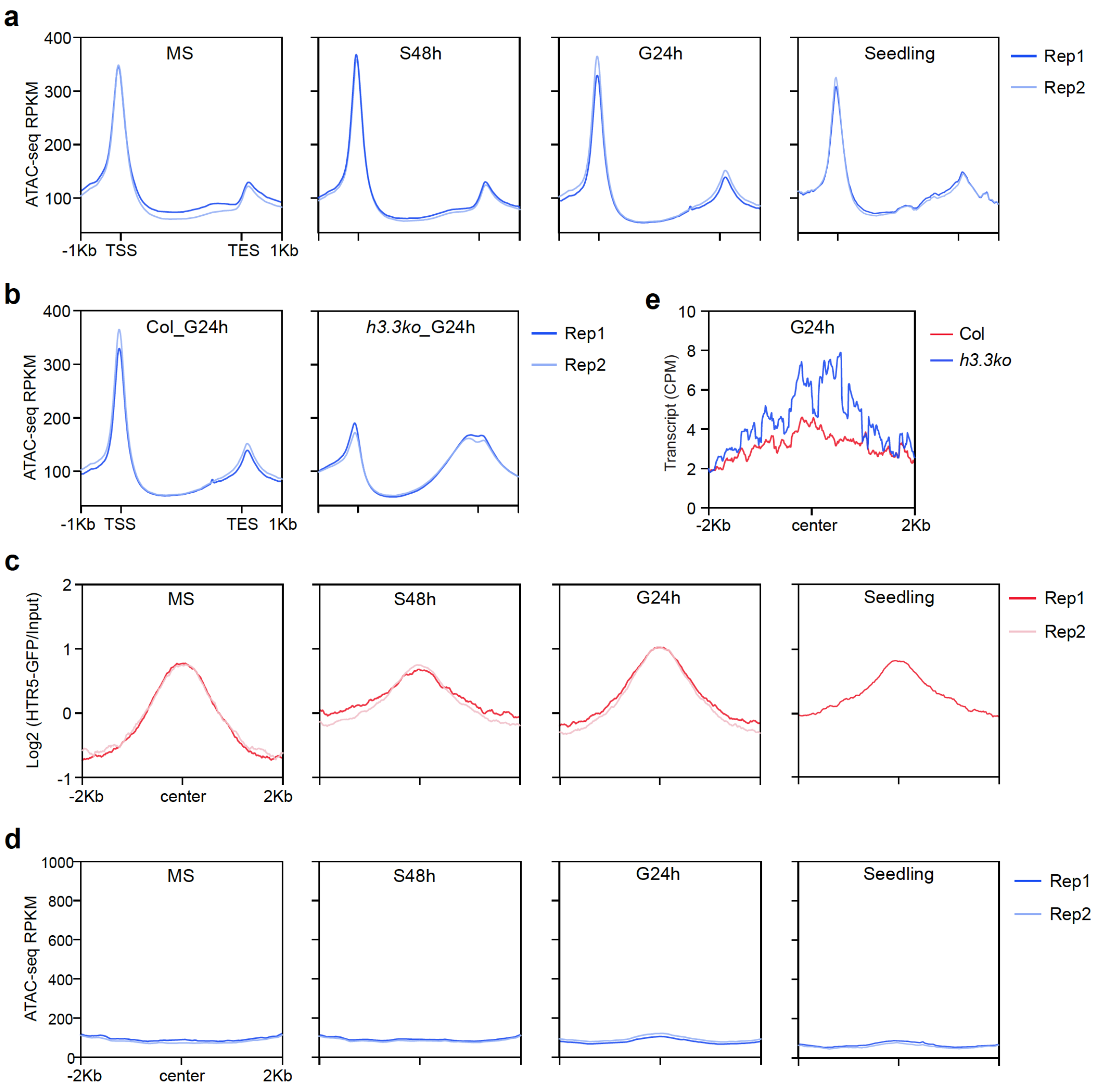

Supplementary Figure 12. Chromatin accessibility analyses during germination and in seedlings.

a. Metaplot of ATAC-seq signals in mature seeds, during germination and in 10-day-old seedlings over all genes.

b. Metaplot of ATAC-seq signals in imbibed Col and *h3.3ko* seeds over all genes.

c and d. Metaplot of HTR5-GFP ChIP-seq (c) and ATAC-seq (d) signals in mature seeds, during germination and in 10-day-old seedlings over accessibility significantly increased regions in mature *h3.3ko* seeds compared with Col.

e. Metaplot of transcripts in imbibed Col and *h3.3ko* seeds over accessibility significantly increased regions in mature *h3.3ko* seeds compared with Col. The profiles were generated after merging three biological replicates.

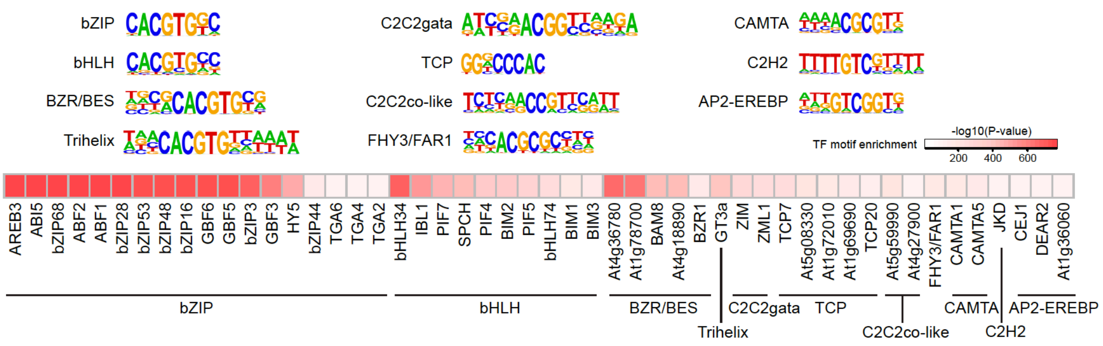

Supplementary Figure 13. Identification of transcription factor binding motifs of at accessibility decreased regions in mature *h3.3ko* seeds compared with Col.

Top 50 transcription factors with their binding motifs enriched in accessibility significantly decreased regions in mature *h3.3ko* seeds compared with Col. The representative DNA binding motif sequences in each class are shown.

Supplementary Table 1. Numbers of mapped reads in RNA-seq, ChIP-seq, ATAC-seq and BS-seq.

Experiment	Sample	Number of mapped reads
**RNA-seq**	Col_MS_1	42,771,255
	Col_MS_2	43,522,042
	Col_MS_3	45,321,666
	Col_S12h_1	42,769,670
	Col_S12h_2	42,068,778
	Col_S12h_3	42,588,357
	Col_S24h_1	42,717,146
	Col_S24h_2	41,514,410
	Col_S24h_3	41,100,658
	Col_S48h_1	42,784,633
	Col_S48h_2	41,746,446
	Col_S48h_3	42,609,198
	Col_G12h_1	41,908,169
	Col_G12h_2	42,843,680
	Col_G12h_3	41,208,944
	Col_G24h_1	41,146,343
	Col_G24h_2	41,493,850
	Col_G24h_3	42,656,327
	h3.3ko_MS_1	44,185,509
	h3.3ko_MS_2	43,951,937
	h3.3ko_MS_3	45,228,773
	h3.3ko_S12h_1	43,165,504
	h3.3ko_S12h_2	43,683,708
	h3.3ko_S12h_3	41,479,732
	h3.3ko_S24h_1	41,637,947
	h3.3ko_S24h_2	42,819,977
	h3.3ko_S24h_3	41,955,739
	h3.3ko_S48h_1	43,223,466
	h3.3ko_S48h_2	42,764,922
	h3.3ko_S48h_3	41,801,537
	h3.3ko_G12h_1	42,449,438
	h3.3ko_G12h_2	42,335,758
	h3.3ko_G12h_3	43,739,988
	h3.3ko_G24h_1	42,159,645
	h3.3ko_G24h_2	42,198,128
	h3.3ko_G24h_3	42,917,352
**Strand-specific RNA-seq**	Col_MS_1	46,625,958
	Col_MS_2	40,150,566
	Col_MS_3	43,471,932
	h3.3ko_MS_1	48,275,889
	h3.3ko_MS_2	44,333,111

	h3.3ko_MS_3	48,006,291
**ChIP-seq**	HTR5-GFP_MS_1_input	10,310,971
	HTR5-GFP_MS_2_input	8,658,993
	HTR5-GFP_MS_1_GFP_IP	11,717,702
	HTR5-GFP_MS_2_GFP_IP	5,932,850
	HTR5-GFP_MS_1_H2A.Z_IP	25,970,238
	HTR5-GFP_MS_2_H2A.Z_IP	31,166,506
	HTR5-GFP_S48h_1_input	7,596,469
	HTR5-GFP_S48h_2_input	13,465,070
	HTR5-GFP_S48h_1_GFP_IP	8,036,824
	HTR5-GFP_S48h_2_GFP_IP	9,416,504
	HTR5-GFP_G24h_1_input	10,607,918
	HTR5-GFP_G24h_2_input	12,504,971
	HTR5-GFP_G24h_1_GFP_IP	10,861,953
	HTR5-GFP_G24h_2_GFP_IP	9,025,282
**ATAC-seq (h3.3ko)**	Col_MS_1	10,308,725
	Col_MS_2	15,788,642
	Col_S48h_1	16,907,549
	Col_S48h_2	14,210,358
	Col_G24h_1	12,656,074
	Col_G24h_2	10,503,110
	Col_Seedling_1	10,672,105
	Col_Seedling_2	9,847,754
	h3.3ko_MS_1	11,563,748
	h3.3ko_MS_2	11,702,975
	h3.3ko_G24h_1	17,519,596
	h3.3ko_G24h_2	16,846,128
**ATAC-seq (h2a.z)**	Col_MS_1	7,213,265
	Col_MS_2	11,247,954
	h2a.z_MS_1	11,146,880
	h2a.z_MS_2	13,746,335
**BS-seq**	Col_MS_1	13,444,622
	Col_MS_2	11,882,854
	h3.3ko_MS_1	7,148,130
	h3.3ko_MS_2	9,620,680

Supplementary Table 2. Primers used in this study.

Experiment	Primer (5' to 3')
**RT-qPCR**	
HTR5	TCGTAAGTCTACTGGAGGAAAG
	ACGCAAGTCAGTCTTGAAATCC
HTR13	GAAATCCACAGGAGGCAAAG
	ACTTCCTGATCTCTCTCAAGGC
GA3OX1	CCTCAACTACTGCGATATCGTTG
	CCATTCAATGTCTTCTTCGCTG
GA3OX2	TCCACGATTTCCGTAAACTCTG
	AATGCGAACCACATCAACTTGG
GA20OX1	ATACTTTCATGGCTCTATCGAAC
	AGCTCTCTCGGTGGCGTCACTAC
GA20OX2	TTCATCGATCTCTCAAGCCAAG
	TGCATAGCCACAACTCTCACC
CYP707A2	ATGGTGGTTGCACTGGAAAGAG
	TGCTCACTAACACCATTCGAGC
PP2A	TCGGATGACGATTCTTCGTGCAG
	CTTGGTCGACTATCGGAATGAGAG
**ChIP-qPCR**	
AT1G36730_I	GTCTGAACAGGCTTCTCTCTTTC
	TCGTGATGTCCTCCTCAAAACGC
AT1G36730_II	CATCGAGTGGCTCCAGAATGC
	AAGACGCACACTGAACCAGACAG
AT3G44110_I	GCGAAAAGATGTTCGGTAGAGG
	ATCAGGATGATTCTTGATAGCGG
AT3G44110_II	GTTTGGGCTCCAACCAATTTGTC
	GTTTCGATGTTCCACCGAAGTGGG
AT5G22000_I	GACGACGTCGGAGGGGCATTTG
	AGTAGAAGGATCGCTTTCGCAG
AT5G22000_II	ATGTCTCTGAAAATCACACTCCTG
	CAGAACCAGTCGCACATGTTTC
