# Supplementary material for: Histone H3.3 deposition in seed is essential for the post-embryonic developmental competence in Arabidopsis
Source: Nat Commun. 2022 Dec 13;13:7728. doi: 10.1038/s41467-022-35509-6 (PMC9747979; doi:10.1038/s41467-022-35509-6)
Supplement: Supplementary file 3 — Description of Additional Supplementary Files [file 41467_2022_35509_MOESM3_ESM.pdf]

Title: Supplementary Data 1.
Description: Transcript level decreased and increased genes in *h3.3ko*.

Title: Supplementary Data 2.
Description: Accessibility significantly decreased and increased regions in *h3.3ko* mature seeds.

Title: Supplementary Data 3.
Description: Genes associated with accessibility significantly decreased and increased regions in *h3.3ko* mature seeds.

Title: Supplementary Data 4.
Description: Enriched TF binding motifs in H3.3-established open chromatin regions.
