# Supplementary material for: Histone H3.3 deposition in seed is essential for the post-embryonic developmental competence in Arabidopsis
Source: Nat Commun. 2022 Dec 13;13:7728. doi: 10.1038/s41467-022-35509-6 (PMC9747979; doi:10.1038/s41467-022-35509-6)
Supplement: Supplementary file 8 — Reporting Summary [file 41467_2022_35509_MOESM8_ESM.pdf]

nature portfolio

Corresponding author(s): Danhua Jiang

Last updated by author(s): Dec 2, 2022

Reporting Summary

Nature Portfolio wishes to improve the reproducibility of the work that we publish. This form provides structure for consistency and transparency in reporting. For further information on Nature Portfolio policies, see our [Editorial Policies] and the [Editorial Policy Checklist].

Statistics

For all statistical analyses, confirm that the following items are present in the figure legend, table legend, main text, or Methods section.

n/a	Confirmed	
☐	☒	The exact sample size (n) for each experimental group/condition, given as a discrete number and unit of measurement
☐	☒	A statement on whether measurements were taken from distinct samples or whether the same sample was measured repeatedly
☐	☒	The statistical test(s) used AND whether they are one- or two-sided<br>*Only common tests should be described solely by name; describe more complex techniques in the Methods section.*
☒	☐	A description of all covariates tested
☐	☒	A description of any assumptions or corrections, such as tests of normality and adjustment for multiple comparisons
☐	☒	A full description of the statistical parameters including central tendency (e.g. means) or other basic estimates (e.g. regression coefficient) AND variation (e.g. standard deviation) or associated estimates of uncertainty (e.g. confidence intervals)
☐	☒	For null hypothesis testing, the test statistic (e.g. F, t, r) with confidence intervals, effect sizes, degrees of freedom and P value noted<br>*Give P values as exact values whenever suitable.*
☒	☐	For Bayesian analysis, information on the choice of priors and Markov chain Monte Carlo settings
☐	☒	For hierarchical and complex designs, identification of the appropriate level for tests and full reporting of outcomes
☒	☐	Estimates of effect sizes (e.g. Cohen's d, Pearson's r), indicating how they were calculated

Our web collection on statistics for biologists contains articles on many of the points above.

Software and code

Policy information about availability of computer code

Data collection	No software was used for data collection
Data analysis	RNA-seq data analysis<br>Adapter trimming was performed and low-quality reads were filtered with fastp version 0.20.1. Reads were mapped to the Arabidopsis genome (TAIR10) using Hisat2 version 2.1.10. Numbers of mapped reads were determined with SAMtools version 1.9. Reads per gene were counted by HTseq version 0.11.2. Transcripts per million (TPM) values were generated using R. Differential gene expression analysis was performed using DESeq2 version 1.26. Genes displayed a more than two-fold expression change and had a P adjust value < 0.05 were considered as differentially expressed. The PCA plot was generated with plotPCA in DESeq2 version 1.26.0. Gene ontology analysis was performed with DAVID. Clustering of genes associated with accessibility decreased regions in mature h3.3ko seeds compared with Col was performed based on their expression changes during germination in Col using K-Means clustering in R.<br>ChIP-seq data analysis<br>Adapter trimming was performed and low-quality reads were filtered with fastp version 0.20.1. Reads were mapped to the Arabidopsis genome (TAIR10) with Botiew2 version 2.4.2 and filtered for duplicated reads by using Picard version 2.24.0 MarkDuplicates. Numbers of mapped reads were determined with SAMtools version 1.9. Heatmaps and average normalized ChIP-seq profiles were generated using deepTools utilities plotHeatmap and plotProfile.<br>ATAC-seq data analysis<br>Adapter trimming was performed and low-quality reads were filtered with fastp version 0.20.1. Reads were mapped to the Arabidopsis genome (TAIR10) with Botiew2 version 2.4.2. Reads mapped to chloroplast and mitochondria genome were removed and duplicated reads were filtered with Picard version 2.24.0 MarkDuplicates. Numbers of mapped reads were determined with SAMtools version 1.9. ATAC-seq open chromatin peaks were identified using MACS2 version 2.1.2 with default parameters. The q-value cutoff for peak calling was 0.05. Only peaks identified from both biological replicates were kept. Peak distributions were analyzed with ChIPseeker version 1.22.1. To identify

differentially enriched peaks, a common peak set was created by merging peaks in Col and h3.3ko, scores for open chromatin peak regions were calculated with deepTools utility multiBigwigSummary, and differential peaks were called by requiring more than two-fold difference and P adjust < 0.05. Genes were assigned to the peaks when the genic and 1kb upstream regions overlapped with the peak regions for at least one base pair. DNA motif analysis was performed with HOMER (version 4.11) using "findMotifs.pl".

BS-seq data analysis

Adapter trimming was performed and low-quality reads were filtered with fastp version 0.20.1. Reads were mapped to the Arabidopsis genome (TAIR10) with BS-Seeker2 version 2.1.8 using default parameters. Duplicated reads were filtered with Picard version 2.24.0 MarkDuplicates. CG, CHG, and CHH methylation levels were calculated with CGmaptools (version 0.1.2). CG DMRs were called with CGmaptools using dmr function with default parameters.

For manuscripts utilizing custom algorithms or software that are central to the research but not yet described in published literature, software must be made available to editors and reviewers. We strongly encourage code deposition in a community repository (e.g. GitHub). See the Nature Portfolio guidelines for submitting code & software for further information.

Data

Policy information about availability of data

All manuscripts must include a data availability statement. This statement should provide the following information, where applicable:

- Accession codes, unique identifiers, or web links for publicly available datasets
- A description of any restrictions on data availability
- For clinical datasets or third party data, please ensure that the statement adheres to our policy

The datasets generated during the current study are available in the GEO repository GSE209645. The private token for data access is olyfyiiafrontkf. The ChIP-seq data of HTR5-GFP in seedlings were downloaded from GEO GSE167384.

Human research participants

Policy information about studies involving human research participants and Sex and Gender in Research.

Reporting on sex and gender	*Use the terms sex (biological attribute) and gender (shaped by social and cultural circumstances) carefully in order to avoid confusing both terms. Indicate if findings apply to only one sex or gender; describe whether sex and gender were considered in study design whether sex and/or gender was determined based on self-reporting or assigned and methods used. Provide in the source data disaggregated sex and gender data where this information has been collected, and consent has been obtained for sharing of individual-level data; provide overall numbers in this Reporting Summary. Please state if this information has not been collected. Report sex- and gender-based analyses where performed, justify reasons for lack of sex- and gender-based analysis.*
Population characteristics	*Describe the covariate-relevant population characteristics of the human research participants (e.g. age, genotypic information, past and current diagnosis and treatment categories). If you filled out the behavioural & social sciences study design questions and have nothing to add here, write "See above."*
Recruitment	*Describe how participants were recruited. Outline any potential self-selection bias or other biases that may be present and how these are likely to impact results.*
Ethics oversight	*Identify the organization(s) that approved the study protocol.*

Note that full information on the approval of the study protocol must also be provided in the manuscript.

Field-specific reporting

Please select the one below that is the best fit for your research. If you are not sure, read the appropriate sections before making your selection.

[x] Life sciences [ ] Behavioural & social sciences [ ] Ecological, evolutionary & environmental sciences

For a reference copy of the document with all sections, see nature.com/documents/nr-reporting-summary-flat.pdf

Life sciences study design

All studies must disclose on these points even when the disclosure is negative.

Sample size	No sample size calculation was performed
Data exclusions	No data exclusion
Replication	Three replicates were performed for RNA-seq, RT-qPCR, ChIP-qPCR and germination test. Two replicates were performed for ChIP-seq, ATAC-seq and BS-seq
Randomization	For all examples, samples were imbibed side by side, each replicate on separate plate

Blinding | No blinding needed

Reporting for specific materials, systems and methods

We require information from authors about some types of materials, experimental systems and methods used in many studies. Here, indicate whether each material, system or method listed is relevant to your study. If you are not sure if a list item applies to your research, read the appropriate section before selecting a response.

Materials & experimental systems

n/a	Involved in the study
☐	☒ Antibodies
☒	☐ Eukaryotic cell lines
☒	☐ Palaeontology and archaeology
☒	☐ Animals and other organisms
☒	☐ Clinical data
☒	☐ Dual use research of concern

Methods

n/a	Involved in the study
☐	☒ ChIP-seq
☒	☐ Flow cytometry
☒	☐ MRI-based neuroimaging

Antibodies

Antibodies used: anti-GFP (Thermo Fisher Scientific, A-11122), anti-H2A.Z (Yelagandula et al., 2014), anti-Pol II (abcam, ab26721)

Validation:
anti-GFP (Thermo Fisher Scientific, A-11122): https://www.thermofisher.cn/cn/zh/antibody/product/GFP-Antibody-Polyclonal/A-11122
anti-H2A.Z (Yelagandula et al., 2014): doi: 10.1016/j.cell.2014.06.006
anti-Pol II (abcam, ab26721): https://www.abcam.com/RNA-polymerase-II-CTD-repeat-YSPTSPS-antibody-ChIP-Grade-ab26721.html

ChIP-seq

Data deposition

☒ Confirm that both raw and final processed data have been deposited in a public database such as GEO.

☒ Confirm that you have deposited or provided access to graph files (e.g. BED files) for the called peaks.

Data access links
May remain private before publication.
https://www.ncbi.nlm.nih.gov/geo/query/acc.cgi?acc=GSE209645

Files in database submission
ChIP-seq
HTR5-GFP_G24h_1_input_R1.fq.gz
HTR5-GFP_G24h_2_input_R1.fq.gz
HTR5-GFP_MS_1_input_R1.fq.gz
HTR5-GFP_MS_2_input_R1.fq.gz
HTR5-GFP_S48h_1_input_R1.fq.gz
HTR5-GFP_S48h_2_input_R1.fq.gz
HTR5-GFP_G24h_1_GFP_IP_R1.fq.gz
HTR5-GFP_G24h_2_GFP_IP_R1.fq.gz
HTR5-GFP_MS_1_GFP_IP_R1.fq.gz
HTR5-GFP_MS_2_GFP_IP_R1.fq.gz
HTR5-GFP_S48h_1_GFP_IP_R1.fq.gz
HTR5-GFP_S48h_2_GFP_IP_R1.fq.gz
HTR5-GFP_MS_1_H2A.Z_IP_R1.fq.gz
HTR5-GFP_MS_2_H2A.Z_IP_R1.fq.gz
HTR5-GFP_G24h_1_input_R2.fq.gz
HTR5-GFP_G24h_2_input_R2.fq.gz
HTR5-GFP_MS_1_input_R2.fq.gz
HTR5-GFP_MS_2_input_R2.fq.gz
HTR5-GFP_S48h_1_input_R2.fq.gz
HTR5-GFP_S48h_2_input_R2.fq.gz
HTR5-GFP_G24h_1_GFP_IP_R2.fq.gz
HTR5-GFP_G24h_2_GFP_IP_R2.fq.gz
HTR5-GFP_MS_1_GFP_IP_R2.fq.gz
HTR5-GFP_MS_2_GFP_IP_R2.fq.gz
HTR5-GFP_S48h_1_GFP_IP_R2.fq.gz
HTR5-GFP_S48h_2_GFP_IP_R2.fq.gz

HTR5-GFP_MS_1_H2A.Z_IP_R2.fq.gz
HTR5-GFP_MS_2_H2A.Z_IP_R2.fq.gz
HTR5-GFP_MS_1_input.bw
HTR5-GFP_MS_2_input.bw
HTR5-GFP_S48h_1_input.bw
HTR5-GFP_S48h_2_input.bw
HTR5-GFP_G24h_1_input.bw
HTR5-GFP_G24h_2_input.bw
HTR5-GFP_MS_1_GFP_IP.bw
HTR5-GFP_MS_2_GFP_IP.bw
HTR5-GFP_S48h_1_GFP_IP.bw
HTR5-GFP_S48h_2_GFP_IP.bw
HTR5-GFP_G24h_1_GFP_IP.bw
HTR5-GFP_G24h_2_GFP_IP.bw
HTR5-GFP_MS_1_H2A.Z_IP.bw
HTR5-GFP_MS_2_H2A.Z_IP.bw
ATAC-seq (h3.3ko)
Col_G24h_1_R1.fq.gz
Col_G24h_2_R1.fq.gz
Col_MS_1_R1.fq.gz
Col_MS_2_R1.fq.gz
Col_S48h_1_R1.fq.gz
Col_S48h_2_R1.fq.gz
Col_Seedling_1_R1.fq.gz
Col_Seedling_2_R1.fq.gz
h3.3ko_MS_1_R1.fq.gz
h3.3ko_MS_2_R1.fq.gz
Col_G24h_1_R2.fq.gz
Col_G24h_2_R2.fq.gz
Col_MS_1_R2.fq.gz
Col_MS_2_R2.fq.gz
Col_S48h_1_R2.fq.gz
Col_S48h_2_R2.fq.gz
Col_Seedling_1_R2.fq.gz
Col_Seedling_2_R2.fq.gz
h3.3ko_MS_1_R2.fq.gz
h3.3ko_MS_2_R2.fq.gz
h3.3ko_G24h_1_R1.fq.gz
h3.3ko_G24h_1_R2.fq.gz
h3.3ko_G24h_2_R1.fq.gz
h3.3ko_G24h_2_R2.fq.gz
Col_MS_1.bw
Col_MS_2.bw
Col_S48h_1.bw
Col_S48h_2.bw
Col_G24h_1.bw
Col_G24h_2.bw
Col_Seedling_1.bw
Col_Seedling_2.bw
h3.3ko_MS_1.bw
h3.3ko_MS_2.bw
h3.3ko_G24h_1.bw
h3.3ko_G24h_2.bw
Accessibility_decreased_in_h3.3ko.bed
Accessibility_increased_in_h3.3ko.bed
ATAC-seq (h2a.z)
Col_MS_1_R1.fq.gz
Col_MS_1_R2.fq.gz
Col_MS_2_R1.fq.gz
Col_MS_2_R2.fq.gz
h2a.z_MS_1_R1.fq.gz
h2a.z_MS_1_R2.fq.gz
h2a.z_MS_2_R1.fq.gz
h2a.z_MS_2_R2.fq.gz
Col_MS_1.bw
Col_MS_2.bw
h2a.z_MS_1.bw
h2a.z_MS_2.bw

Genome browser session
(e.g. UCSC)

Provide a link to an anonymized genome browser session for "Initial submission" and "Revised version" documents only, to enable peer review. Write "no longer applicable" for "Final submission" documents.

Methodology

Replicates

ATAC-seq: two replicates
HTR5-GFP ChIP-seq: two replicates
H2A.Z ChIP-seq: two replicates

Sequencing depth

All sequencing reads are paired-end 150bp, listed are the numbers of uniquely mapped reads
ChIP-seq
HTR5-GFP_MS_1_input 10,310,971
HTR5-GFP_MS_2_input 8,658,993
HTR5-GFP_MS_1_GFP_IP 11,717,702
HTR5-GFP_MS_2_GFP_IP 5,932,850
HTR5-GFP_MS_1_H2A.Z_IP 25,970,238
HTR5-GFP_MS_2_H2A.Z_IP 31,166,506
HTR5-GFP_S48h_1_input 7,596,469
HTR5-GFP_S48h_2_input 13,465,070
HTR5-GFP_S48h_1_GFP_IP 8,036,824
HTR5-GFP_S48h_2_GFP_IP 9,416,504
HTR5-GFP_G24h_1_input 10,607,918
HTR5-GFP_G24h_2_input 12,504,971
HTR5-GFP_G24h_1_GFP_IP 10,861,953
HTR5-GFP_G24h_2_GFP_IP 9,025,282
ATAC-seq (h3.3ko)
Col_MS_1 10,308,725
Col_MS_2 15,788,642
Col_S48h_1 16,907,549
Col_S48h_2 14,210,358
Col_G24h_1 12,656,074
Col_G24h_2 10,503,110
Col_Seedling_1 10,672,105
Col_Seedling_2 9,847,754
h3.3ko_MS_1 11,563,748
h3.3ko_MS_2 11,702,975
h3.3ko_G24h_1 17,519,596
h3.3ko_G24h_2 16,846,128
ATAC-seq (h2a.z)
Col_MS_1 7,213,265
Col_MS_2 11,247,954
h2a.z_MS_1 11,146,880
h2a.z_MS_2 13,746,335

Antibodies

anti-GFP (Thermo Fisher Scientific, A-11122), anti-H2A.Z (Yelagandula et al., 2014)

Peak calling parameters

system('cat call_peak.txt | while read id; do sample_name=${id%.re*}; ~/anaconda2/bin/macs2 callpeak -t $id -f BAMPE -g 1.2e8 -q 0.01 --max-gap 150 --call-summits -n ${sample_name} 2> ${sample_name}".log"; done')
system('cat call_peak.txt | while read id1 id2; do sample_name1=${id1%%.*}; sample_name2=${id2%%.*}; ~/anaconda2/bin/macs2 callpeak -t $id1 -c $id2 --broad --broad-cutoff 0.05 -f BAMPE -g 1.2e8 -n ${sample_name1}"vs"${sample_name2} 2> ${sample_name1}"vs"${sample_name2}".log"; done')

Data quality

ATAC-seq
Col_MS_1: 7225 peaks
Col_MS_2: 11269 peaks
h3.3ko_MS_1: 14356 peaks
h3.3ko_MS_2: 17189 peaks
ChIP-seq
HTR5-GFP_MS_1 (anti-GFP): 14741 peaks
HTR5-GFP_MS_2 (anti-GFP): 11904 peaks
Only peaks identified from both biological replicates were kept.

Software

fastp version 0.20.1
Botiew2 version 2.4.2
Picard version 2.24.0
SAMtools version 1.9
MACS2 version 2.1.2
